# Psychomotor Development in Pediatric Patients with Congenital Heart Defects Prior to Surgical Intervention: Findings from a Prospective Cross-Sectional Study

**DOI:** 10.3390/medicina62010156

**Published:** 2026-01-13

**Authors:** Lacramioara Eliza Chiperi, Cristina Tecar, Radu Samuel Pop

**Affiliations:** 1 Department of Pediatric Cardiology, Emergency Institute for Cardiovascular Diseases and Heart Transplant, 540136 Targu Mures, Romania; lacramioara-eliza.pop@umfst.ro; 2 RoNeuro Institute, Centre for Neurological Research and Diagnostic, 400364 Cluj-Napoca, Romania; cristina.pantelemon@umfcluj.ro; 3 3rd Department of Pediatrics, “Iuliu Hațieganu” University of Medicine and Pharmacy, 400217 Cluj-Napoca, Romania

**Keywords:** psychomotor development, congenital heart defect, Denver Developmental Screening Test II, unrepaired congenital heart defect, cyanosis, neurodevelopment

## Abstract

*Background and Objectives:* Psychomotor developmental delay is a frequent comorbidity in children with congenital heart defects (CHD), especially after surgical correction of the CHD and exposure to risk factors such as anesthesia, cardiopulmonary bypass and postoperative complications. Yet psychomotor delay is present in these patients before surgical correction but is under-recognized. Evidence focusing solely on unrepaired CHD remains limited. *Materials and Methods*: This prospective cross-sectional study evaluated 153 and included 77 children under 6 years of age with unrepaired CHD, stratified into cyanotic (*n* = 31) and non-cyanotic (*n* = 46) CHD, admitted to a pediatric cardiology department over a period of 5 years. Psychomotor development was assessed using the Denver Developmental Screening Test II (DDST-II), standardized for pediatric population. Associations with clinical, perinatal, and demographic factors were analyzed using univariate and multivariate methods. *Results*: Developmental delay was identified in 97% of cyanotic and 54% of non-cyanotic patients. Compared to healthy norms, CHD patients had significantly lower global developmental scores (*p* = 0.03). Gross motor and personal-social domains were most frequently affected. Prenatal CHD diagnosis correlated with better global developmental scores (*p* = 0.012), and breastfeeding was associated with improved outcomes compared with formula or mixed feeding (*p* = 0.008). *Conclusions*: Infants and young children with CHD are at increased risk of early psychomotor developmental delay, particularly in the gross motor and personal–social domains, even before exposure to surgical or intensive care damaging factors. Systematic psychomotor surveillance, integration of protective factors such as prenatal diagnosis and breastfeeding, and timely access to multidisciplinary interventions are essential to optimize long-term outcomes in this vulnerable population.

## 1. Introduction

Congenital heart defects (CHDs) are the most common congenital malformations, affecting 0.8–1.2% of live births worldwide [1]. Beyond their cardiovascular implications, CHD exerts a substantial impact on brain development and long-term neurodevelopmental outcomes, with psychomotor delay representing one of the most frequent and clinically relevant comorbidities [2,3,4]. Psychomotor development integrates motor coordination, movement planning, sensory processing, language, and social interaction, and early disturbances in these domains may compromise functional independence, school readiness, and quality of life across the lifespan.

Despite increasing awareness of neurodevelopmental morbidity in CHDs, developmental surveillance remains inconsistently implemented in routine clinical care. In Europe, no unified guidelines exists for systematic neurodevelopmental monitoring of children with CHD, and recent data indicate that fewer than half of pediatric cardiology centers provide structured developmental follow-up programs [5]. This contrasts with American Heart Association recommendations, which emphasize regular neurodevelopmental screening both before and after cardiac surgery [2].

The mechanisms underlying psychomotor delay in children with CHD are multifactorial and begin early in life. Genetic vulnerability, altered fetal cerebral blood flow, impaired oxygen and substrate delivery to the developing brain, and delayed cortical maturation have all been implicated [4,5,6,7,8,9,10,11,12]. These intrinsic factors are particularly relevant in complex and cyanotic forms of CHD, where chronic hypoxemia and hemodynamic instability during critical periods of brain development may disrupt white matter maturation and cortical connectivity, thereby increasing vulnerability to motor and cognitive impairment [9].

Although perioperative factors such as cardiopulmonary bypass, anesthesia exposure, and postoperative complications are well-established contributors to adverse neurodevelopmental outcomes, growing evidence suggests that developmental vulnerability may already be present before surgical correction [7]. Some studies have reported preoperative motor and cognitive deficits in infants with CHD, indicating that the cardiac lesion itself—and its physiological consequences—may play an independent role in shaping early psychomotor trajectories [8,9,10]. However, most available data continue to focus on postoperative populations, making it difficult to disentangle lesion-related effects from those associated with surgical and intensive care exposure.

Across existing literature, gross motor and personal–social domains appear particularly susceptible to early impairment in children with CHD [13]. In addition to biological mechanisms, environmental and contextual factors may further influence early development. Prior to surgical intervention, infants with CHD often experience restricted opportunities for motor exploration, reduced tolerance for physical activity, prolonged hospitalization, and heightened caregiver anxiety, all of which may limit sensorimotor stimulation and social engagement during sensitive developmental windows.

In parallel, potentially modifiable protective factors may mitigate early developmental risk. Prenatal diagnosis of CHD has been associated with optimized perinatal management, delivery planning in specialized centers, earlier stabilization, and improved parental preparedness, which together may support more favorable early developmental trajectories. Breastfeeding has also been consistently linked to improved neurocognitive and psychomotor outcomes in the general pediatric population and may represent an additional protective factor in children with CHD through its nutritional, neurobiological, and relational benefits. Nevertheless, the extent to which these factors influence psychomotor development specifically in unrepaired CHD remains insufficiently characterized.

Despite these observations, data focusing specifically on psychomotor development in children with unrepaired CHD remain scarce, and preoperative developmental screening is not routinely integrated into clinical practice in many European centers [5]. As a result, early developmental delays may go unrecognized at a stage when targeted interventions are most effective. Early interventions initiated during infancy—such as physiotherapy, occupational therapy, and caregiver-centered developmental guidance—are associated with improved long-term motor, cognitive, and behavioral outcomes [8]. A clearer characterization of baseline psychomotor functioning before surgical exposure is therefore essential to inform surveillance strategies and guide early multidisciplinary care in this high-risk population.

The present prospective cross-sectional study aims to evaluate psychomotor development in children under six years of age with unrepaired CHD using the Denver Developmental Screening Test II (DDST-II). We further explore domain-specific developmental patterns and examine clinical, perinatal, and demographic factors potentially associated with psychomotor outcomes. By focusing on the preoperative period, this study seeks to clarify baseline neurodevelopmental vulnerability in CHD and to support the integration of early developmental surveillance into routine congenital cardiology care.

## 2. Materials and Methods

### 2.1. Study Design and Setting

We conducted a prospective cross-sectional study in a pediatric cardiology department of a tertiary center, over a five-year recruitment period (2020–2024). The study design was chosen to evaluate psychomotor development in children with unrepaired CHD before exposure to surgical or intensive care–related factors.

### 2.2. Ethical Considerations

The study protocol received approval from the institutional ethics committees of both the hospital and the affiliated university of medicine. Written informed consent was obtained from parents or legal guardians of patients prior to inclusion.

### 2.3. Participants

Eligible participants were children under 6 years of age with a confirmed diagnosis of CHD who had not undergone surgical or interventional correction and had no previously documented psychomotor delay. CHD diagnosis was established by pediatric cardiologists using clinical examination, electrocardiography, and echocardiography, supplemented by advanced imaging (e.g., CT scan) when indicated.

Prior to inclusion, a brief clinical developmental screening was conducted by a pediatrician. Children exhibiting neurological deficits or previously diagnosed developmental disorders were excluded. Additional exclusion criteria included: known genetic syndromes, perinatal asphyxia (Apgar score < 7 at 5 min with arterial pH < 7 or base excess > 12 mmol/L), prematurity (<36 weeks gestation), families and children whose primary language was not Romanian, and institutionalized children.

Healthy Romanian children from the 2012 national DDST-II standardization cohort served as the reference group.

Sample size was calculated assuming a 10% prevalence of psychomotor delay in the CHD population, a 95% confidence level, and a 5% margin of error, based on national pediatric population data revealing a CHD incidence of 2.1% in Romania [14,15].

### 2.4. Data Collection

Clinical, demographic, and paraclinical data were collected prospectively during hospitalization and entered into a structured electronic database (Table 1). Personal and family history were obtained via structured interviews with the child’s mother or legal guardian.

Participants were stratified based on peripheral oxygen saturation (SpO_2_) into cyanotic (SpO_2_ < 94%) and non-cyanotic (SpO_2_ ≥ 94%) groups, using 94% as the clinical threshold for adequate oxygenation [16]. SpO_2_ was measured via non-invasive pulse oximetry. Laboratory data of patients was also included in the database.

### 2.5. Psychomotor Development Assessment

Psychomotor development was assessed preoperatively using the DDST-II, standardized for the Romanian pediatric population in 2012 [17]. The test evaluates four developmental domains: personal–social, fine motor–adaptive, language and gross motor.

A single examiner, trained and certified in DDST-II administration, conducted all assessments to minimize inter-rater variability. The evaluation lasted 20–40 min and included direct observation of how the child accomplished age-related tasks and a structured parental interview. The DDST-II result was classified as normal, suspect, or untestable. Patients with suspect results were further addressed to pediatric neurology for confirmation of the developmental delay and adequate interventions.

In order to obtain more information about development, scoring was improved by following the modified method described by Ware et al. [18] to better reflect developmental trajectories in children with complex medical conditions. The method included calculation of supplementary scores: basal level = highest item passed among three consecutive items; passing level = highest item passed before any failed item; high level = highest item passed before three consecutive failures; top level = highest item attempted beyond failure.

For each domain, a domain-specific developmental score was calculated as the mean of basal and high scores. A global developmental score was also calculated as the mean of the domain-specific developmental scores. A developmental score of 1.0 indicated age-appropriate development, >1.0 indicated advanced development, and <1.0 indicated delay.

To explore potential age-related differences in psychomotor development, participants were additionally stratified into age groups reflecting key developmental stages: infants ≤12 months, toddlers 13–36 months, and preschool children > 36 months.

Both global psychomotor development scores and domain-specific scores (personal–social, fine motor, language, and gross motor) were analyzed across age groups. Comparisons were performed using one-way ANOVA or Kruskal–Wallis tests, as appropriate. This exploratory analysis aimed to assess whether the prevalence and pattern of abnormal psychomotor development varied according to age at assessment.

### 2.6. Statistical Analysis

Data were analyzed using Stata 13 and GraphPad InStat. Normality was tested with the Shapiro–Wilk test. Depending on distribution, continuous variables were compared using Student’s *t*-test or Mann–Whitney U test, and categorical variables using the Chi-square test.

Associations between developmental scores and categorical predictors were assessed via one-way ANOVA, with Bonferroni correction for multiple comparisons. Correlations were examined using linear regression. Logistic regression was applied for multivariate adjustment of potential confounders. Outliers were identified by interquartile range (IQR) method and replaced with the median; missing values (< 10% per variable) were imputed using the mean. *p* value ≤ 0.05 was considered statistically significant.

## 3. Results

### 3.1. Study Population

**Figure 1:**
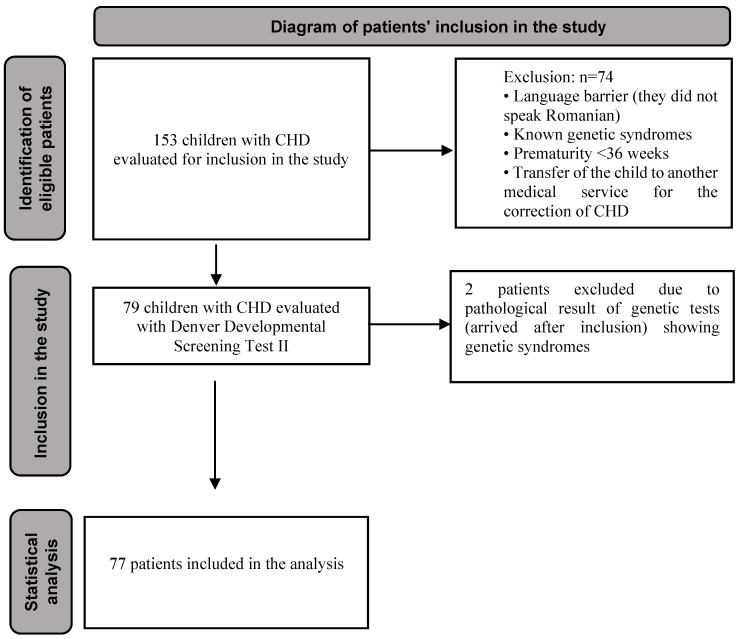
Flowchart of participant recruitment and inclusion in the study. The diagram illustrates patient screening, eligibility assessment, and final inclusion in the prospective cross-sectional analysis. CHD = congenital heart defect.

Out of 153 children admitted to the pediatric cardiology department during the five-year study period, 77 met the inclusion criteria and were enrolled (Figure 1).

**Figure 2:**
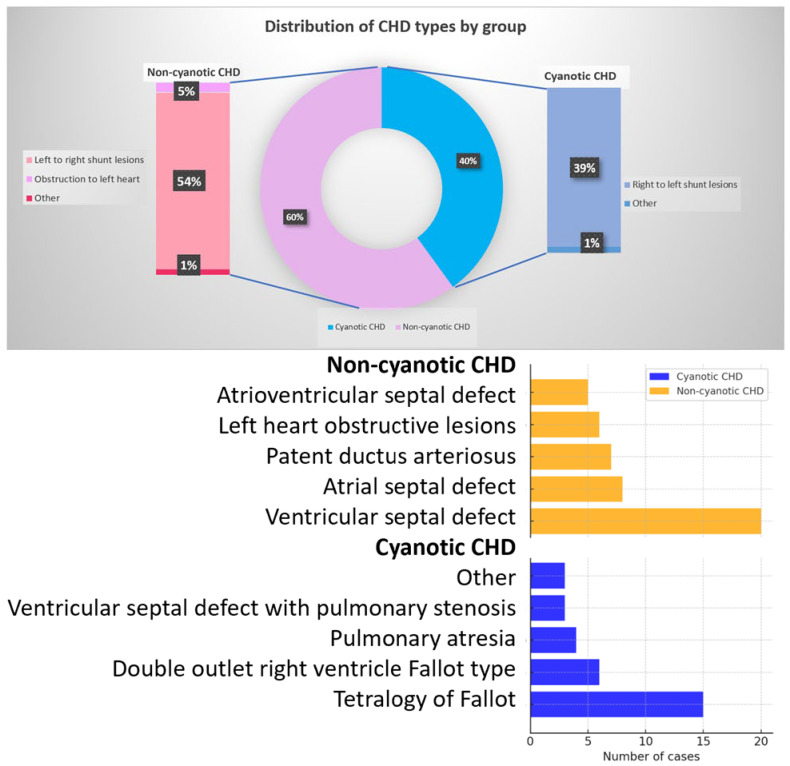
Distribution of congenital heart defect (CHD) types in the study cohort. Cyanotic CHD included right-to-left shunt lesions, while non-cyanotic CHD consisted predominantly of left-to-right shunt defects. Percentages represent the proportion of each CHD subtype within the respective group. CHD = congenital heart defect.

The cohort included 46 children with non-cyanotic CHD (59.7%) and 31 with cyanotic CHD (40.3%). As illustrated in Figure 2, non-cyanotic CHD was predominantly represented by left-to-right shunt lesions (atrial septal defect, ventricular septal defect, atrioventricular septal defect, persistent ductus arteriosus, aorto-pulmonary window), whereas cyanotic CHD consisted almost exclusively of right-to-left shunt lesions (tetralogy of Fallot, double outlet right ventricle, Fallot type, pulmonary atresia) with similar hemodynamic characteristics. This distribution supports the clinical comparability within each group while highlighting fundamental physiological differences between cyanotic and non-cyanotic lesions.

The median age at assessment was 9 months (IQR: 8 months), with infants (<12 months) comprising 58% of the sample.

Gender distribution was balanced (52% male, 48% female). More than half of the participants (57%) came from rural areas.

### 3.2. Baseline Characteristics

As summarized in Table 1, children with cyanotic CHD had significantly lower peripheral oxygen saturation and higher hemoglobin levels compared with the non-cyanotic group, reflecting chronic hypoxemia. No significant differences were observed in demographic or perinatal characteristics between groups.

### 3.3. Psychomotor Developmental Outcomes

**Figure 3:**
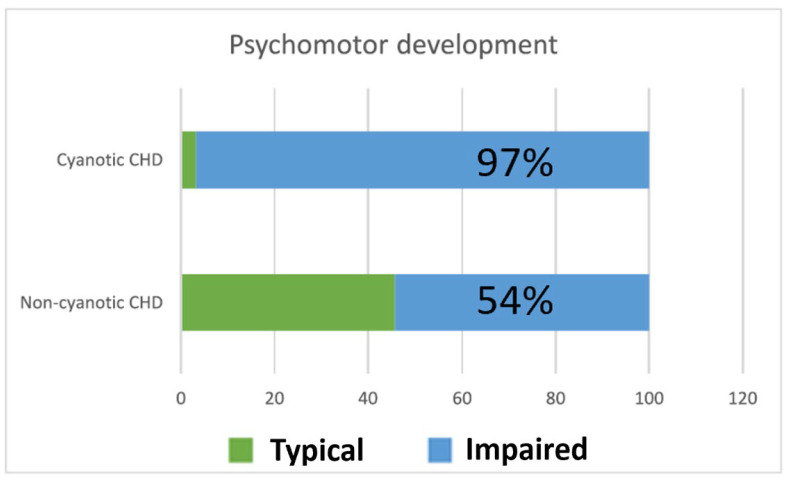
Prevalence of psychomotor developmental delay in children with congenital heart defects. Bars represent the percentage of children classified as having psychomotor delay according to DDST-II results. CHD = congenital heart defect. DDST-II = Denver Developmental Screening Test. Comparison between groups was performed using the Chi-square test.

Compared to the age-specific normative reference values of the DDST-II, children with CHD had significantly lower global developmental scores (*p* = 0.03). Figure 3 highlights a markedly higher prevalence of psychomotor developmental delay in children with cyanotic CHD compared with those with non-cyanotic lesions. Nearly all children in the cyanotic group exhibited developmental delay, whereas just over half of the non-cyanotic group were affected, emphasizing the substantial neurodevelopmental burden associated with cyanosis.

Table 2 presents domain-specific developmental scores, showing consistently lower mean scores in children with cyanotic CHD across all domains. Although these differences did not reach statistical significance, gross motor and personal–social domains exhibited the greatest numerical disparities.

**Figure 4:**
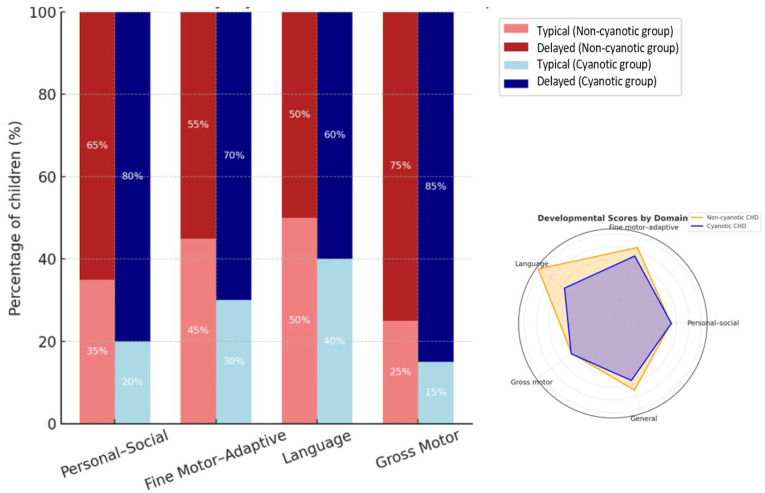
Domain-specific prevalence of psychomotor developmental delay in children with congenital heart defects. Developmental delay was assessed across gross motor, fine motor, language, and personal–social domains using the DDST-II. Bars indicate the proportion of children with delay in each domain. CHD = congenital heart defect; DDST-II = Denver Developmental Screening Test II.

As shown in Figure 4, gross motor and personal–social domains were the most frequently affected across both CHD groups. While developmental delay was observed in all domains, these findings indicate a disproportionate vulnerability of motor and social functioning in early childhood among patients with CHD.

**Figure 5:**
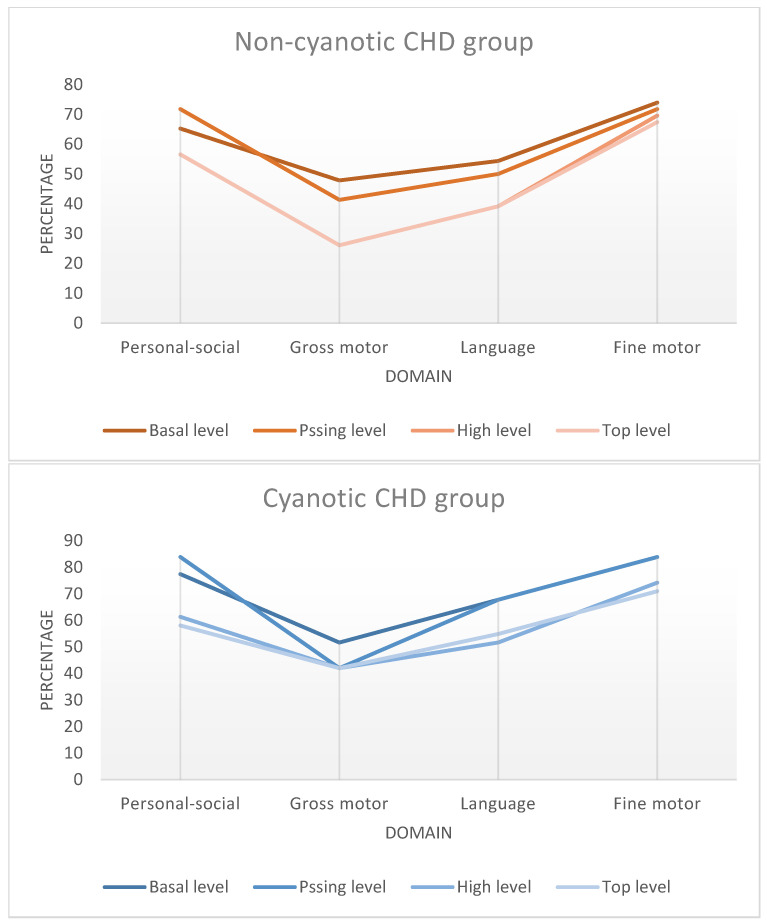
Distribution of developmental performance levels across psychomotor domains in children with congenital heart defects. Performance was categorized according to DDST-II scoring criteria. Gross motor domain showed the lowest performance levels across both cyanotic and non-cyanotic CHD groups. CHD = congenital heart defect; DDST-II = Denver Developmental Screening Test II.

Figure 5 demonstrates that, in both cyanotic and non-cyanotic CHD, the lowest performance was consistently observed in the gross motor domain across developmental levels, with progressively better performance in language and fine motor domains. This pattern suggests domain-specific developmental vulnerability rather than a uniform global delay.

### 3.4. Factors Associated with Psychomotor Development

**Figure 6:**
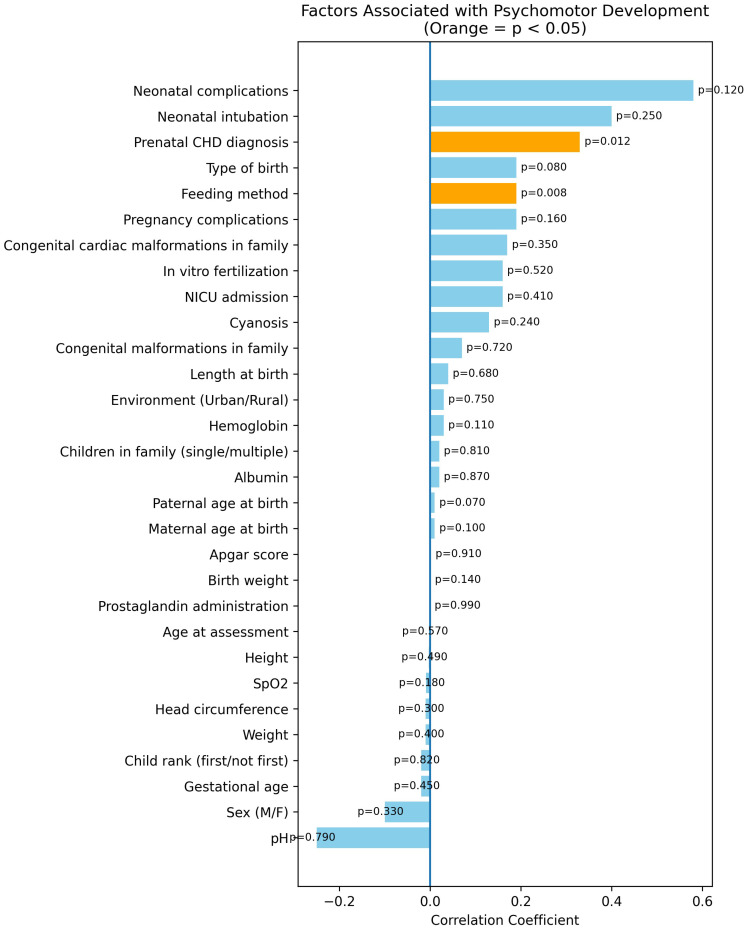
Factors associated with global psychomotor developmental score in children with congenital heart defects. Forest plot displays regression coefficients and 95% confidence intervals derived from multivariable linear regression analysis. Prenatal diagnosis of CHD and exclusive breastfeeding were significantly associated with higher global developmental scores. Statistical significance was defined as *p* < 0.05. CHD = congenital heart defect; CI = confidence interval.

Correlation analysis identified two factors significantly associated with higher developmental scores: prenatal CHD diagnosis (correlation coefficient 0.33; *p* = 0.012) and feeding method (exclusive breastfeeding associated with better scores; correlation coefficient 0.19; *p* = 0.008) as illustrated in Figure 6.

Additionally, socio-economic factors, including employment status (maternal *p* = 0.49; paternal *p* = 1), higher-level education (maternal *p* = 0.05; paternal *p* = 0.14), and access to early intervention services (*p* = 0.45), showed no significant association with psychomotor development in our study.

### 3.5. Age-Stratified Psychomotor Development

**Figure 7:**
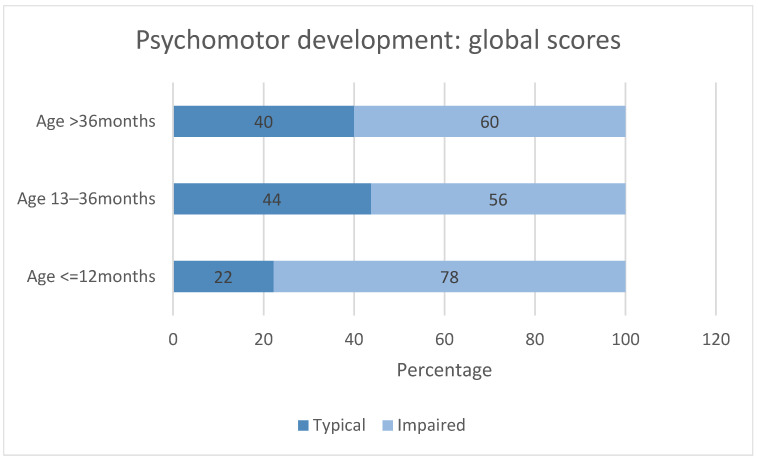

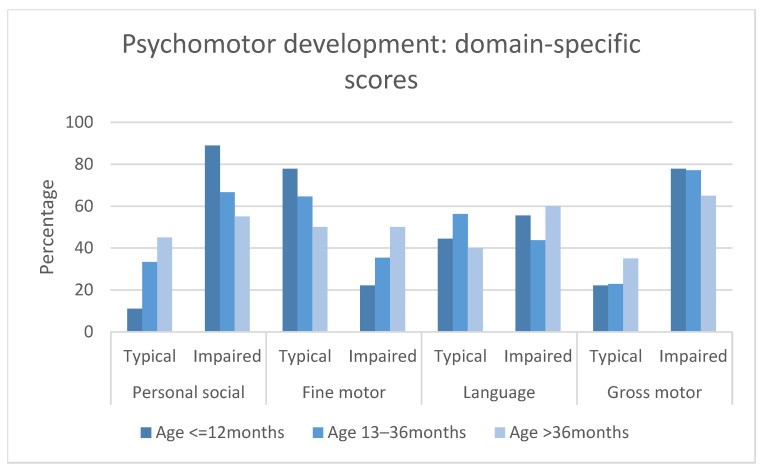
Age-related patterns of psychomotor global and domain-specific development in children with congenital heart defects. Children were stratified into three age categories (≤12 months, 13–36 months, and >36 months). Bars represent the percentage of children classified as having typical or impaired psychomotor development within each age group according to Denver Developmental Screening Test results. The upper panel illustrates the proportion of children with typical and impaired global psychomotor development. The lower panel presents domain-specific psychomotor outcomes (personal–social, fine motor, language, and gross motor) stratified by age group. Percentages represent the distribution of typical and impaired scores within each domain and age category. Impaired psychomotor development was observed across all age categories, with the highest prevalence in infants ≤ 12 months.

When stratified by age, an important percentage of abnormal global psychomotor development was observed across all age groups. Infants younger than 12 months showed the highest prevalence of global abnormal development, compared with older children as can be seen in Figure 7.

Analysis of domain-specific scores revealed age-related differences in the pattern of impairment (Figure 7). In infants ≤ 12 months, impairment was particularly prominent in the personal–social, fine motor, and gross motor domains. In children aged 13–36 months, impairments remained frequent across all domains, with notable involvement of fine motor and gross motor skills. In the oldest age group (>36 months), although the overall proportion of impaired children remained high, a relative shift toward language and gross motor difficulties was observed.

Overall, while the proportion of typical global psychomotor development tended to increase with age, domain-specific impairments persisted across all developmental stages. Differences between age groups did not reach statistical significance for global scores (*p* = 0.48) or for domain-specific scores like personal-social (*p* = 0.2), fine motor (*p* = 0.31), language (*p* = 0.44), and gross motor (*p* = 0.56), likely due to the limited sample size within each group.

## 4. Discussion

### 4.1. Main Findings

This prospective, cross-sectional study demonstrates that psychomotor developmental delay is highly prevalent in children with unrepaired congenital heart defects, even before exposure to surgical or intensive care–related factors. Nearly all children with cyanotic CHD and more than half of those with non-cyanotic lesions showed evidence of developmental delay, underscoring that neurodevelopmental vulnerability emerges early in the disease course. By focusing exclusively on the preoperative period, our findings help isolate the intrinsic contribution of the cardiac defect and its physiological consequences to early psychomotor development.

Although children with cyanotic CHD consistently showed lower developmental scores across domains, intergroup differences did not reach statistical significance, likely reflecting limited sample size. Nevertheless, the observed pattern aligns with prior reports linking chronic hypoxemia and hemodynamic instability to adverse neurodevelopmental outcomes in CHD.

### 4.2. Psychomotor Developmental Screening and Assessment

Psychomotor development was assessed using the DDST-II, a widely used screening instrument for children under six years of age across gross motor, fine motor, language, and personal–social domains [19]. In Romania, the DDST-II has undergone national standardization and cultural–linguistic adaptation, supporting its applicability in the present cohort [20]. As with all screening tools, the DDST-II does not provide a definitive diagnosis but rather identifies children at risk who may benefit from further evaluation and early intervention.

To improve sensitivity in this medically complex population, we applied an adapted scoring approach based on the method described by Ware et al. [18], which allows a more nuanced characterization of developmental trajectories across domains. This approach is particularly relevant in children with CHD, whose development may not follow typical patterns due to medical vulnerability, reduced physical activity, and early life stress.

### 4.3. Patterns of Psychomotor Developmental Delay

Consistent with previous literature [21,22,23,24,25,26,27,28,29,30,31,32,33], gross motor and personal–social domains emerged as the most affected areas in our cohort. These domains depend heavily on adequate cerebral oxygen delivery [34,35], muscular endurance, and opportunities for exploration and social interaction, all of which may be compromised in infants with CHD [22,23,24]. Similar domain-specific vulnerabilities have been reported in both preoperative and postoperative CHD populations [36,37,38,39,40,41,42,43].

Interestingly, children with cyanotic CHD demonstrated marginally higher fine motor and language scores compared with their non-cyanotic peers, a finding also noted in earlier studies [31]. This pattern may reflect compensatory mechanisms, whereby increased caregiver engagement or structured interaction supports certain developmental domains despite physiological disadvantage. Such findings highlight the complexity of developmental adaptation in children with CHD and caution against assuming uniform impairment across domains.

The age-stratified analysis of both global and domain-specific psychomotor scores indicates that developmental vulnerability in children with unrepaired congenital heart defects is present from early infancy and persists throughout childhood. The very high prevalence of global impairment observed during the first year of life highlights a critical period of susceptibility, likely related to rapid neurodevelopmental demands combined with limited physiological reserve. Domain-specific analysis further suggests that the profile of psychomotor impairment changes with age rather than resolving completely. Early infancy is characterized predominantly by personal–social and motor difficulties, whereas older children exhibit a broader distribution of impairments, including language and gross motor domains. Although a modest increase in typical global development was observed with advancing age, more than half of children older than 36 months continued to demonstrate psychomotor impairment, arguing against complete developmental catch-up. These findings emphasize the need for early, repeated, and domain-sensitive neurodevelopmental assessment, as well as timely intervention strategies initiated in infancy and continued across childhood.

### 4.4. Factors Influencing Psychomotor Development

Two potentially modifiable factors—prenatal diagnosis and breastfeeding—were associated with more favorable psychomotor outcomes in our cohort. Prenatal detection of CHD may facilitate optimized perinatal management, early stabilization, and improved parental preparedness, thereby creating a more supportive developmental environment from birth. These findings are consistent with prior evidence linking prenatal diagnosis to improved early outcomes through coordinated care pathways [44].

Breastfeeding was also associated with higher developmental scores, in line with extensive evidence demonstrating its neurocognitive and psychomotor benefits [45,46,47,48,49,50]. Beyond its nutritional advantages, breastfeeding promotes parent–infant bonding and sensory interaction, which may be particularly important for infants with CHD who face early medical challenges. Although direct evidence in CHD populations remains limited, our findings support breastfeeding as a potentially protective factor worthy of further investigation.

Socioeconomic variables, including parental education and employment status, were not significantly associated with psychomotor outcomes in our cohort [51,52]. Nevertheless, the direction of observed trends aligns with broader literature suggesting that parental education, enriched home environments and caregiver resources can positively influence neurodevelopment [53,54]. Larger studies may be better positioned to clarify the contribution of these contextual factors.

### 4.5. Strengths and Limitations

The strengths of this study include its prospective design, standardized developmental assessment, and exclusive focus on the preoperative period, which minimizes confounding from surgical and intensive care exposures. The use of an adapted scoring approach further enhances the sensitivity of psychomotor assessment in this high-risk population.

Several limitations should be acknowledged. The single-center design and modest sample size limit generalizability and statistical power, particularly for subgroup analyses. Neuroimaging correlates of development could not be evaluated due to concerns related to sedation in young children. Finally, as a screening-based study, our findings indicate developmental risk rather than definitive diagnoses.

### 4.6. Clinical and Research Implications

These findings highlight the importance of integrating psychomotor developmental screening into preoperative care for children with CHD. Early identification of at-risk infants provides an opportunity for timely referral to physiotherapy, occupational therapy, and caregiver-focused interventions, potentially mitigating long-term developmental consequences.

Future research should prioritize longitudinal designs to examine developmental trajectories across the perioperative period and to evaluate the effectiveness of early, pre-surgical interventions. Establishing standardized neurodevelopmental surveillance protocols may contribute to more consistent care and improved outcomes for children with congenital heart defects.

## 5. Conclusions

Infants and young children with congenital heart disease are at high risk for psychomotor developmental delay, with gross motor and personal–social domains emerging as the most vulnerable, even prior to surgical intervention. These findings emphasize that neurodevelopmental compromise in CHD arises early, independent of perioperative factors, and highlight the importance of systematic developmental surveillance as an integral component of cardiology care. Age-stratified analyses further demonstrate that abnormal psychomotor development is already highly prevalent in infancy and persists across later developmental stages, with domain-specific patterns evolving over time rather than complete developmental catch-up.

Early identification of at-risk children through structured screening programs—combined with timely access to targeted interventions such as physiotherapy, occupational therapy, and caregiver-centered support—offers an opportunity to mitigate long-term deficits. The absence of standardized European guidelines remains a critical gap, underscoring the need for harmonized, evidence-based frameworks that align with existing U.S. recommendations.

By reinforcing the importance of early protective factors such as prenatal diagnosis, and breastfeeding, this study contributes to the growing body of evidence advocating for multidisciplinary and proactive approaches aimed at optimizing developmental outcomes in children with CHD.

## Figures and Tables

**Table 1:** Baseline demographic, perinatal, and clinical characteristics of children with congenital heart defects. Values are presented as mean ± standard deviation or number (percentage), as appropriate. Comparisons between cyanotic and non-cyanotic CHD groups were performed using Student’s *t*-test or Mann–Whitney U test for continuous variables and Chi-square or Fisher’s exact test for categorical variables. CHD = congenital heart defect; SpO_2_ = peripheral oxygen saturation.

Characteristic	Non-Cyanotic CHD (*n* = 46)	Cyanotic CHD (*n* = 31)	*p*-Value	Missing (*n*)
**Personal and Family History**				
Congenital malformations in family (*n*, %)	3 (6.5)	3 (9.7)	0.61	0
Congenital cardiac malformations in family (*n*, %)	4 (8.7)	4 (12.9)	0.55	0
Maternal age at birth (years, mean ± SD)	28.4 ± 5.7	28.7 ± 6.4	0.82	0
Paternal age at birth (years, mean ± SD)	31.9 ± 5.2	33.2 ± 8.6	0.40	0
Firstborn child (*n*, %)	25 (54.3)	11 (35.5)	0.10	0
In vitro fertilization (*n*, %)	3 (6.5)	1 (3.2)	0.52	1
Prenatal CHD diagnosis (*n*, %)	12 (26.1)	25 (80.6)	0.14	1
Pregnancy complications (*n*, %)	8 (17.4)	8 (25.8)	0.37	1
Urban origin (*n*, %)	21 (45.7)	13 (41.9)	0.74	0
**Neonatal Characteristics**				
Gestational age (weeks, mean ± SD)	38.2 ± 1.6	38.4 ± 1.9	0.40	2
Apgar score at 5 min (median)	9	9	0.23	1
Birth weight (g, mean ± SD)	3049 ± 459	3123 ± 706	0.58	0
Birth length (cm, mean ± SD)	50.1 ± 6.0	50.6 ± 5.0	0.74	7
Delivery method: vaginal (*n*, %)	18 (39.1)	15 (48.4)	0.42	0
NICU admission (*n*, %)	6 (13.0)	10 (32.3)	0.04	0
Neonatal complications (*n*, %)	7 (15.2)	4 (12.9)	0.82	1
Neonatal intubation (*n*, %)	0 (0)	2 (6.5)	0.07	1
Feeding: breast milk only (*n*, %)	14 (30.4)	13 (41.9)	0.38	0
**Clinical Characteristics**				
Male sex (*n*, %)	26 (56.5)	14 (45.2)	0.32	0
Age at evaluation (months, mean ± SD)	12.5 ± 15.0	10.9 ± 6.0	0.33	0
Weight (kg, mean ± SD)	7.84 ± 3.6	8.06 ± 1.9	0.08	0
Height (cm, mean ± SD)	70.0 ± 14.9	72.0 ± 6.9	0.26	0
Head circumference (cm, mean ± SD)	43.6 ± 4.6	45.1 ± 5.1	0.13	0
SpO_2_ (%)	97 ± 1	83 ± 7	<0.0001	0
**Paraclinical Characteristics**				
Hemoglobin (g/dL, mean ± SD)	11.7 ± 1.4	14.1 ± 2.7	<0.0001	1
Albumin (g/dL, mean ± SD)	4.4 ± 0.4	4.3 ± 0.4	0.39	14
Arterial pH (mean ± SD)	7.40 ± 0.05	7.40 ± 0.05	0.07	4

**Table 2:** Domain-specific developmental scores in children with congenital heart defects. Values are presented as mean ± standard deviation. DS = developmental score; CHD = congenital heart defect. Comparisons between cyanotic and non-cyanotic groups were performed using Student’s *t*-test or Mann–Whitney U test, as appropriate.

Domain	Non-Cyanotic CHD (*n* = 46)	Cyanotic CHD (*n* = 31)	*p*-Value
	Mean ± SD	Mean ± SD	
Fine motor–adaptive DS	0.91 ± 0.40	0.92 ± 0.30	0.83
Personal–social DS	1.25 ± 0.70	1.11 ± 0.30	0.81
Gross motor DS	1.45 ± 1.00	0.94 ± 0.30	0.14
Language DS	0.80 ± 0.30	0.81 ± 0.20	0.93
**General DS**	1.10 ± 0.60	0.94 ± 0.20	0.35

## Data Availability

The data presented in this study are available on request from the corresponding author. The data are not publicly available due to privacy and ethical restrictions.
